# A simple liquid chromatography-tandem mass spectrometry method to accurately determine the novel third-generation EGFR-TKI naquotinib with its applicability to metabolic stability assessment

**DOI:** 10.1039/c8ra09812c

**Published:** 2019-02-07

**Authors:** Haitham Alrabiah, Adnan A. Kadi, Mohamed W. Attwa, Ali S. Abdelhameed

**Affiliations:** Department of Pharmaceutical Chemistry, College of Pharmacy, King Saud University P. O. Box 2457 Riyadh 11451 Kingdom of Saudi Arabia halrabiah@ksu.edu.sa akadi@ksu.edu.sa mzeidan@ksu.edu.sa asaber@ksu.edu.sa +966 1146 76 220 +966 1146 70237; Students’ University Hospital, Mansoura University Mansoura 35516 Egypt

## Abstract

Naquotinib (ASP8273, NQT) is a novel third-generation epidermal growth factor receptor tyrosine kinase inhibitor (EGFR-TKIs). NQT was found to be more effective than osimertinib against the EGFR L858R plus T790M mutation (L858R+T790M). A rapid resolution liquid chromatography (RRLC)-tandem mass spectrometry (MS/MS) method was developed and validated for NQT quantification and its metabolic stability was investigated. NQT and foretinib (FTB) as an internal standard (IS) were separated using a mobile phase under isocratic conditions with a C18 column (reversed phase system). The linearity of the analytical method ranged from 5 to 500 ng mL^−1^ (coefficient of correlation [*r*^2^] ≥ 0.9999) in a human liver microsome (HLM) matrix. The limit of detection and limit of quantification were 0.78 and 2.36 ng mL^−1^, respectively. The inter-day and intra-day accuracy and precision were −6.36 to 1.88 and 0.99 to 2.58%, respectively. The metabolic stability of NQT in the HLM matrix was calculated using the *in vitro* half-life (*t*_1/2_, 67.96 min) and intrinsic clearance (Cl_int_, 2.12 mL min^−1^ kg^−1^). NQT is considered to be a moderate extraction ratio drug that is moderately excreted from the human body compared with other related TKIs. This proposed methodology is thought to be the first method for assessing NQT concentration and its metabolic stability.

## Abstract 2

The first established LC-MS/MS method for NQT analysis. NQT was shown to be moderately excreted from the human body.

## Graphical abstract

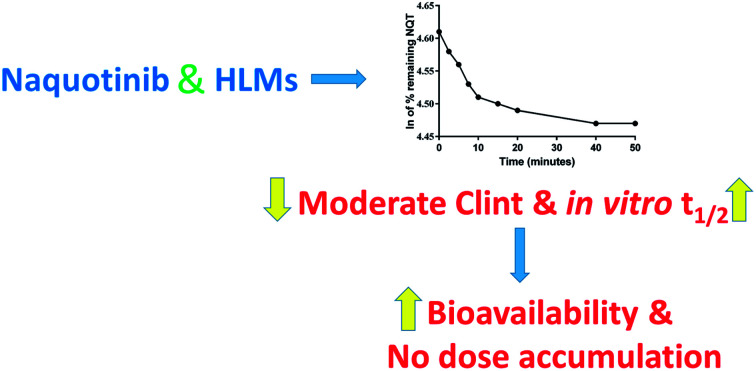

## 1. Introduction

Non-small cell lung cancer (NSCLC) is a common and widespread subgroup of lung cancers^1–5^ and it has an incidence of 90% of all patients suffering from lung cancer. The epidermal growth factor receptor (EGFR) signaling pathway attained considerable importance a few years ago as a therapeutic target in NSCLC.^6^ Tyrosine kinase inhibitors (TKIs) that control the EGFR are very efficacious in EGFR mutation treatment with a characteristic therapeutic window. First-line TKIs controlling EGFR (*e.g.* erlotinib and gefitinib) are characterized by an initially good response against these mutations.^7,8^ Unfortunately, acquired resistance in approximately 60% of patients and the toxicities associated with treatment^9,10^ have decreased their therapeutic efficacies.^11,12^ This encouraged scientists to develop second-generation irreversible EGFR TKIs (*e.g.*, dacomitinib and avitinib).^13,14^ However, NSCLC was shown to acquire resistance to first- and second-generation EGFR-TKIs in approximately 1 year.^15^ The EGFR T790M mutation is found in approximately 50% of NSCLCs resistant to first- and second-generation EGFR-TKIs. The second-generation EGFR TKIs (*e.g.* dacomitinib and avitinib) address the drawbacks of first-line TKIs in the first year from the beginning of treatment.^8,13^

The third-generation TKIs maintain the second-generation drugs' benefits by preventing EGFR mutations and surmounting the T790M resistance mutation.^13,14^ Naquotinib (ASP8273, NQT) is considered a novel third-generation EGFR-TKI and was found to be more effective than osimertinib against the L858R plus T790M mutation (L858R+T790M). Additionally, naquotinib and osimertinib showed a wide therapeutic window and similar efficacy for EGFR exon 20 insertions cells.^16^

A review of the literature proved there were no published chromatographic methods for NQT assay; therefore, we were prompted to establish an LC-MS/MS methodology for this target. The estimation of bioavailability provides an idea of the compound metabolism. If the test drug is quickly metabolized, it will show a low *in vivo* bioavailability value.^17^ NQT could be considered a moderate extraction ratio drug and, hence, is moderately excreted from the human body compared to other TKIs.^18–21^ This implies a lower risk of drug accumulation inside the body in comparison to other TKIs (*e.g.* dacomitinib). Therefore, the metabolic stability of NQT was determined by computing two important parameters (intrinsic clearance and *in vitro* half-life [*t*_1/2_]) that could be utilized to further calculate other physiological parameters (*e.g.*, *in vivo t*_1/2_, bioavailability and hepatic clearance).

## 2. Experimental

### 2.1. Reagents and chemicals

Naquotinib (99.12%) and foretinib (99.81%) were procured from MedChemExpress (USA). Acetonitrile (ACN, HPLC grade), formic acid (HCOOH), ammonium formate (NH_4_COOH) and human liver microsomes, pooled (HLMs, M0567) were procured from Sigma-Aldrich (USA). HPLC grade water (H_2_O) was prepared by a filtration system (Milli-Q Plus, USA).

### 2.2. LC-MS/MS methodology

All LC-MS/MS parameters were optimized to attain the best chromatographic separation of NQT and FTB (IS) with high resolution (Table 1). FTB was selected as the IS in the NQT analysis because the same extraction method could be utilized for both substances (NQT and FTB recoveries were 98.61 ± 2.42% and 98.7 ± 0.7%, respectively, in the HLM matrix) and the elution time of FTB is comparable to that of NQT. The supposed method is fast with a short run time (4 min). Both FTB and NQT are TKIs and were not co-administered to patients, so this method could be utilized for clinical applications, such as pharmacokinetics or therapeutic drug monitoring (TDM), for patients under naquotinib treatment.

**Table 1:** Analytical parameters^a^

LC			MS	
RRLC	Agilent 1200		MS	Agilent 6410 QQQ
Isocratic mobile phase	45% ACN		ESI	Positive ESI
	10 mM NH_4_COOH	55%		Drying gas: N_2_ of low purity at 12 L min^−1^, pressure (60 psi)
		pH: 4.2		
	Flow rate: 0.2 mL min^−1^			
	Injection volume: 1 μL			
Agilent Eclipse plus C_18_ column	100 mm long			Source *T*: 350 °C
	2.1 mm internal diameter			Capillary *V*: 4000 V
	1.8 μm particle size		Collision cell	N_2_ (high purity)
	*T*: 20 ± 2 °C.		Mode	MRM
Mass spectra segment	0.0 to 1.2 min	Flow to waste	Analyte: naquotinib (NQT)	*m*/*z* 563 → *m*/*z* 463, FV^b^: 125 V, CE^c^: of 22 eV
	1.2 to 2.7 min	NQT MRM		*m*/*z* 563 → *m*/*z* 323, FV: 130 V, CE: of 18 eV
	2.7 to 4.0 min	FTB MRM	Foretinib (IS)	*m*/*z* 633 → *m*/*z* 128, FV: 145 V, CE: 20 eV

a LC, liquid chromatography; MS, mass spectrometry.

b Fragmentor voltage.

c Collision energy.

**Scheme 1:**
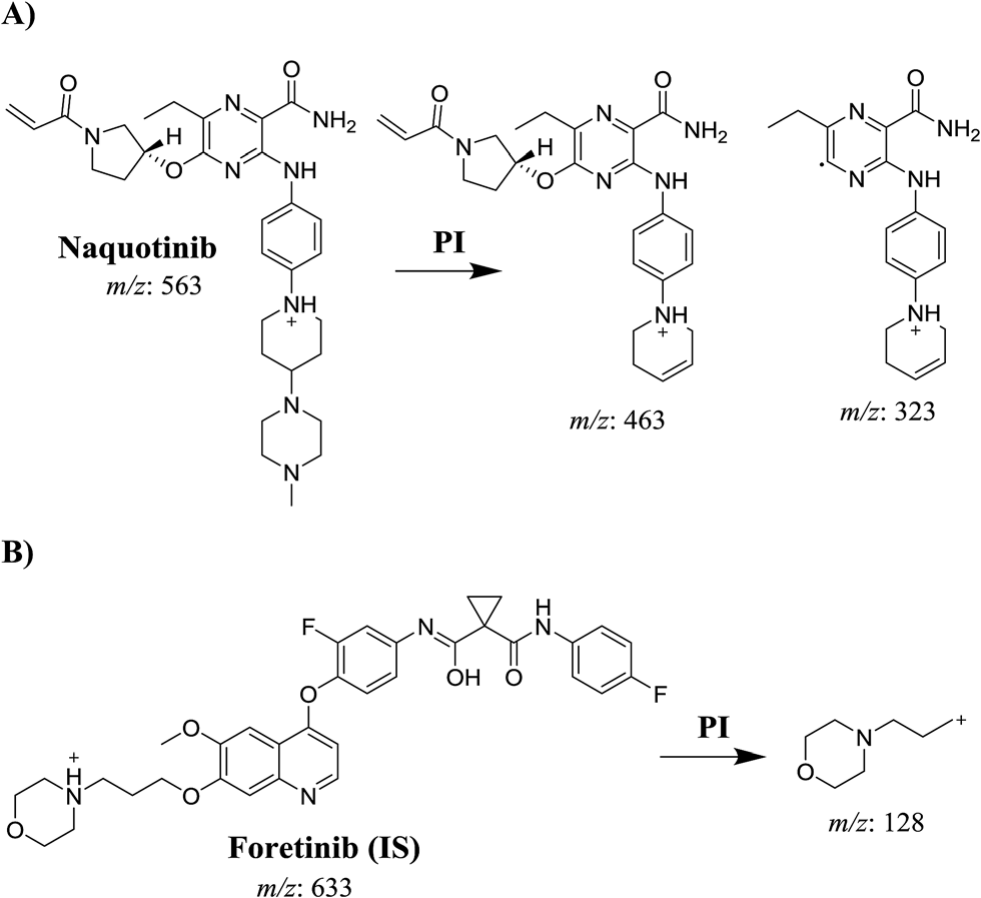


**Fig. 1:**
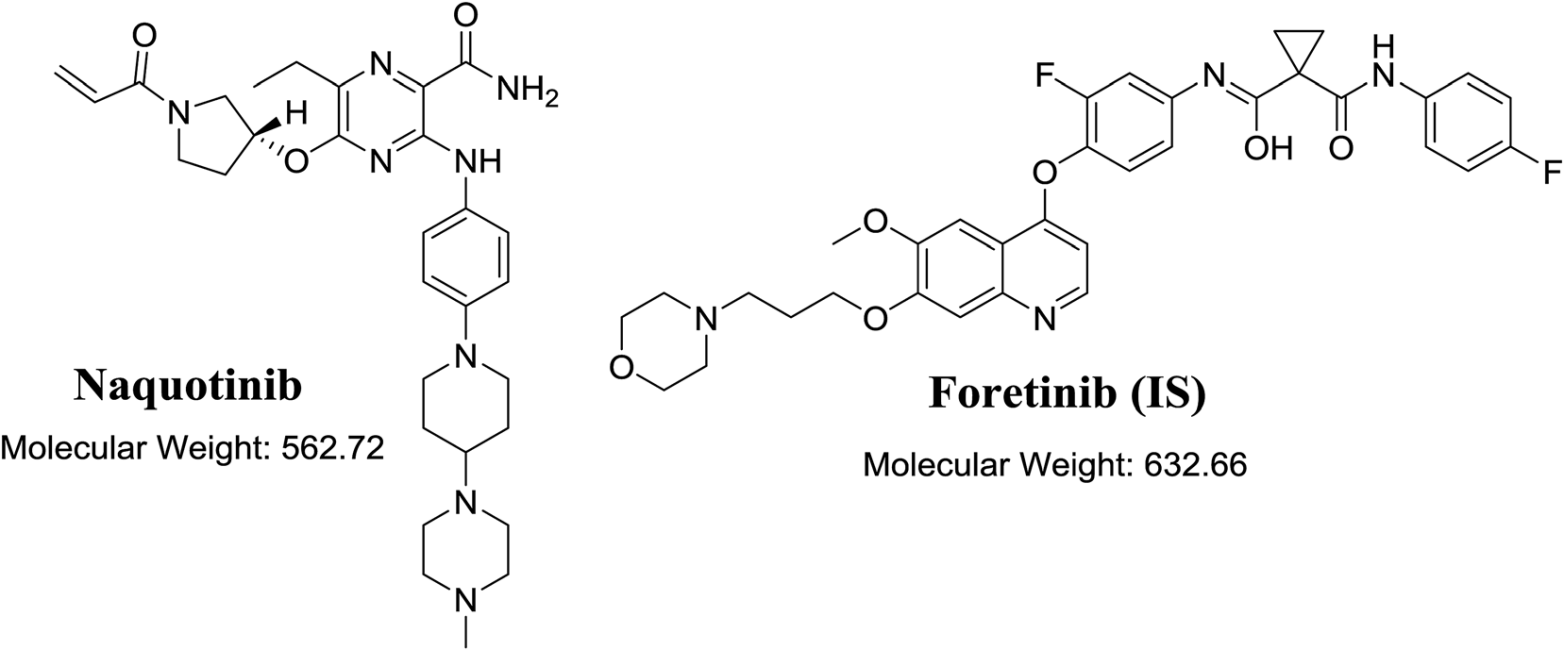
Chemical structure of naquotinib and foretinib (internal standard [IS]).

**Fig. 2:**
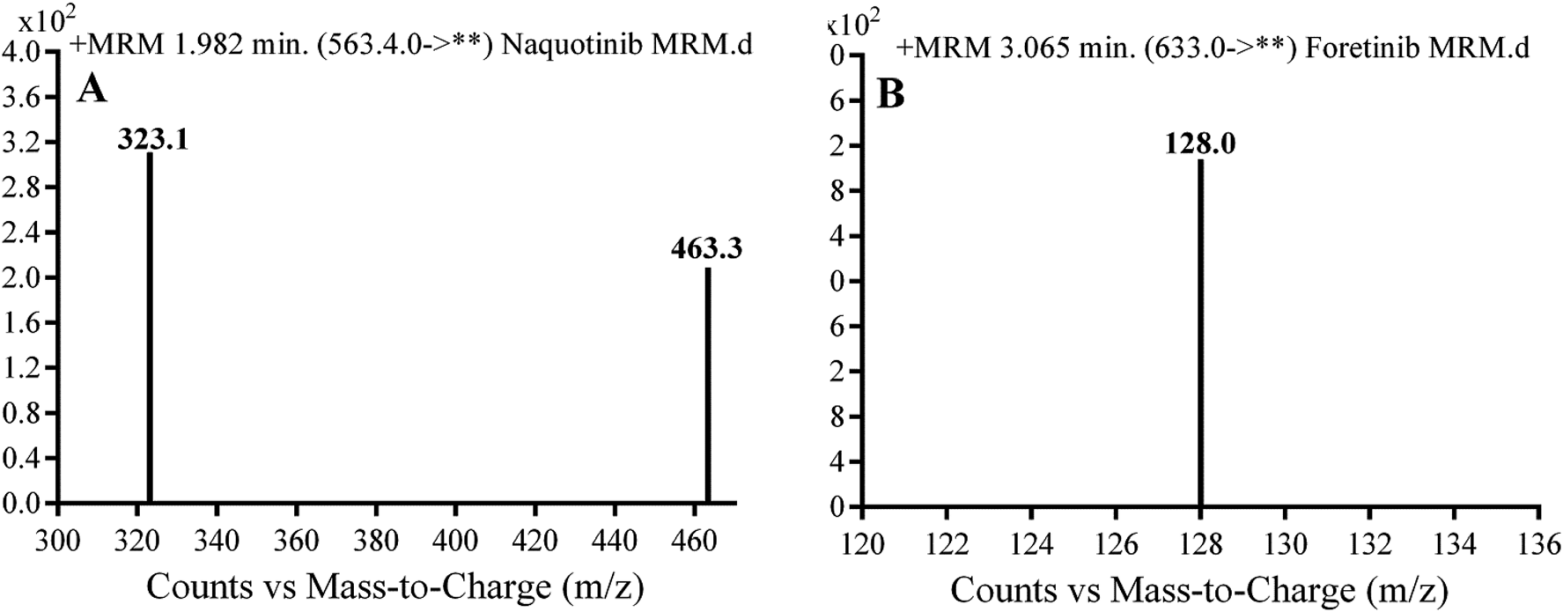
Multiple reaction mode (MRM) mass spectral transitions of (A) naquotinib (NQT) and (B) foretinib (IS).

A triple quadrupole (QqQ) mass spectrometer with an electrospray ionization source interface (ESI, positive mode) was utilized for detection. Low-purity nitrogen (N_2_, 12 L min^−1^) was utilized as the drying gas in the ESI source while high-purity N_2_ (55 psi) was used as the collision gas. The ESI temperature (*T*) and capillary voltage (*V*) were set at 350 °C and 3500 V, respectively. The instruments and data acquisition were controlled using Mass Hunter software. NQT was quantified using the analyzer mode of multiple reaction monitoring (MRM) for the mass reactions (parent to fragment ions) from 563 → 463 and 563 → 323 for NQT and 633 → 128 for FTB (Scheme 1). The fragmentor voltage (FV) was adjusted to 125 and 130 V with collision energies (CEs) of 22 and 18 for NQT, and an FV of 145 V with CE of 20 for FTB. The MRM mass analyzer mode was utilized for NQT quantification to erase any interference of the HLM constituents and to elevate the sensitivity of the proposed LC-MS/MS method (Fig. 1 and 2).

### 2.3. NQT calibration stock solutions

Both NQT and FTB are soluble in dimethyl sulfoxide (DMSO). NQT (2 mg mL^−1^ in DMSO) was diluted 10-fold in the mobile phase to make working NQT solution 1 (WK1, 200 μg mL^−1^), which was further diluted to prepare WK2 (20 μg mL^−1^). FTB (100 μg mL^−1^ in DMSO) was diluted 50-fold in the mobile phase to make WK3 (1 μg mL^−1^).

### 2.4. Preparation of NQT calibration standards

WK2 was mixed with a specific HLM matrix (40.00 μL in 1 mL of phosphate buffer) to establish a calibration curve of 12 standards: 5.00, 10.00, 15.00, 30.00, 50.00, 80.00, 100.00, 150.00, 200.00, 300.00, 400.00, and 500.00 ng mL^−1^. Four concentration levels, 5, 15, 150, and 400 ng mL^−1^, were selected as the lower limit quality control (LLQC), low quality control (LQC), medium quality control (MQC), and high quality control (HQC), respectively. NQT and FTB were extracted using an acetonitrile (ACN) protein precipitation method for the metabolic stability experiments.^22^ ACN (2 mL) was added to each milliliter of the standard solution, followed by centrifugation (14 000 rpm for 12 min) in a thermostatic centrifuge at 4 °C to precipitate proteins and refine the sample to remove unwanted materials. Then, 1 mL of each supernatant was collected and filtered using a syringe filter (0.22 μm pore size). WK3 (50 μL) was added, followed by transference of the mixtures to 1.5 mL vials. The injection volume was 1 μL to facilitate the adjustment of the peak shape by enhancing the sharpness. Control samples were similarly prepared as described above, using the stated phosphate buffer without the HLM matrix to verify that the components of HLM did not interfere at NQT and FTB retention times. A standard calibration curve was constructed by plotting the peak area ratio of NQT to FTB (*y*-axis) against NQT nominal values (*x*-axis). A linear regression equation was utilized to verify the linearity of the supposed method. The slope, intercept, and coefficient of correlation (*r*^2^) values were computed.

### 2.5. Method validation

Parameters for validation of the supposed LC-MS/MS method for NQT assay were described in detail in our previous articles.^21–23^ The following validation parameters, linearity, assay recovery, sensitivity, reproducibility, specificity, limit of quantification (LOQ), and limit of detection (LOD), were utilized for the computation. All validation parameters were computed for NQT. The least squares statistical method was utilized for statistical calculation of the calibration curve equations (*y* = *ax* + *b*). A linear fit was verified by the *r*^2^, which showed linearity in the range of 5–500 ng mL^−1^. The recovery of NQT in the spiked HLM matrix was 98.61 ± 2.42% (relative standard deviation [RSD] < 2.68%).

### 2.6. Metabolic stability of NQT

The NQT metabolic stability was determined by assessing the NQT concentration remaining after incubation with HLMs. Briefly, 1 μM of NQT was incubated with HLMs (1 mg microsomal protein per 1 mL phosphate buffer) and the experiment was repeated three times for confirmation of the results. The metabolic reaction was performed in phosphate buffer (pH 7.4) containing magnesium chloride (MgCl_2_, 3.3 mM). The mixture was pre-incubated for 10 min in a thermostated water bath (37 °C) and then the metabolic reactions were initiated and subsequently stopped by adding NADPH (1 mM) and 2 mL ACN, respectively, at specific time intervals: 0, 2.5, 5, 7.5, 10, 15, 20, 40, 50, and 70 min. The metabolic stability curve for NQT was subsequently constructed.

## 3. Results and discussion

### 3.1. HPLC-MS/MS methodology

Chromatographic conditions, including mobile phase pH and composition, and the stationary phase, were optimized. The pH of the aqueous part (10 mM ammonium formate) was adjusted to 4.2 using formic acid because at a higher pH, peak tailing and an excessive increase in retention time were observed. The ratio between the aqueous and organic parts (ACN) was set to 55 : 45%, because an increase in the ACN ratio resulted in overlapping chromatographic peaks with bad peak resolution, whereas a decrease in the ratio increased the elution time. Various types of stationary phases such as HILIC columns were tested, but NQT and FTB were not retained and good results were finally attained using a C18 column. MRM was used for NQT quantification to remove any HLM matrix interference and enhance the sensitivity of the proposed LC-MS/MS method (Fig. 2).

**Fig. 3:**
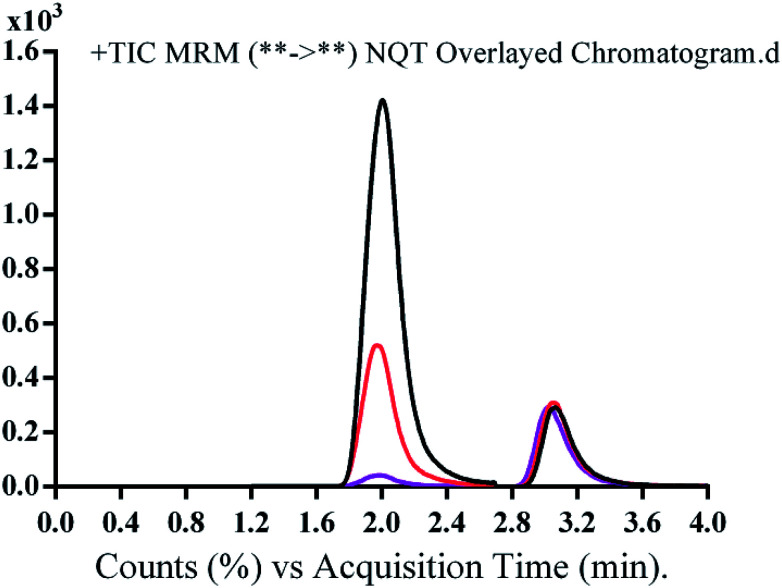
Overlayed multiple reaction mode (MRM) chromatograms of quality control (QC) standards of naquotinib (NQT, 15, 150, and 400 ng mL^−1^) and FTB (50 ng mL^−1^).

The run time for complete elution of NQT and FTB was 4 min with good resolution. There was no carryover in the blank HLM matrix sample. Fig. 3 shows the NQT QC standards as overlaid MRM chromatograms.

### 3.2. Validation of the LC-MS/MS method

#### 3.2.1. Specificity

**Fig. 4:**
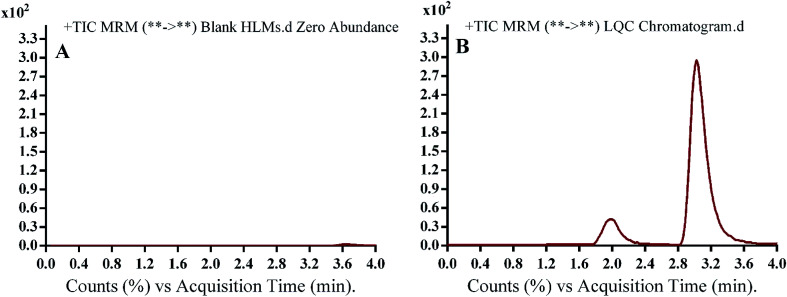
Multiple reaction monitoring (MRM) chromatograms of (A) blank human liver microsomes (HLMs) and (B) naquotinib (NQT, 15 ng mL^−1^, low quality control [LQC]). Blank HLM matrix revealed no matrix interference.

Fig. 4 shows good separation of the NQT and FTB peaks and the lack of any peak in the blank HLM matrix at their retention times, which proved the specificity of the established method. No carryover effect of NQT or FTB was seen in the MRM chromatograms.

#### 3.2.2. Sensitivity and linearity

**Fig. 5:**
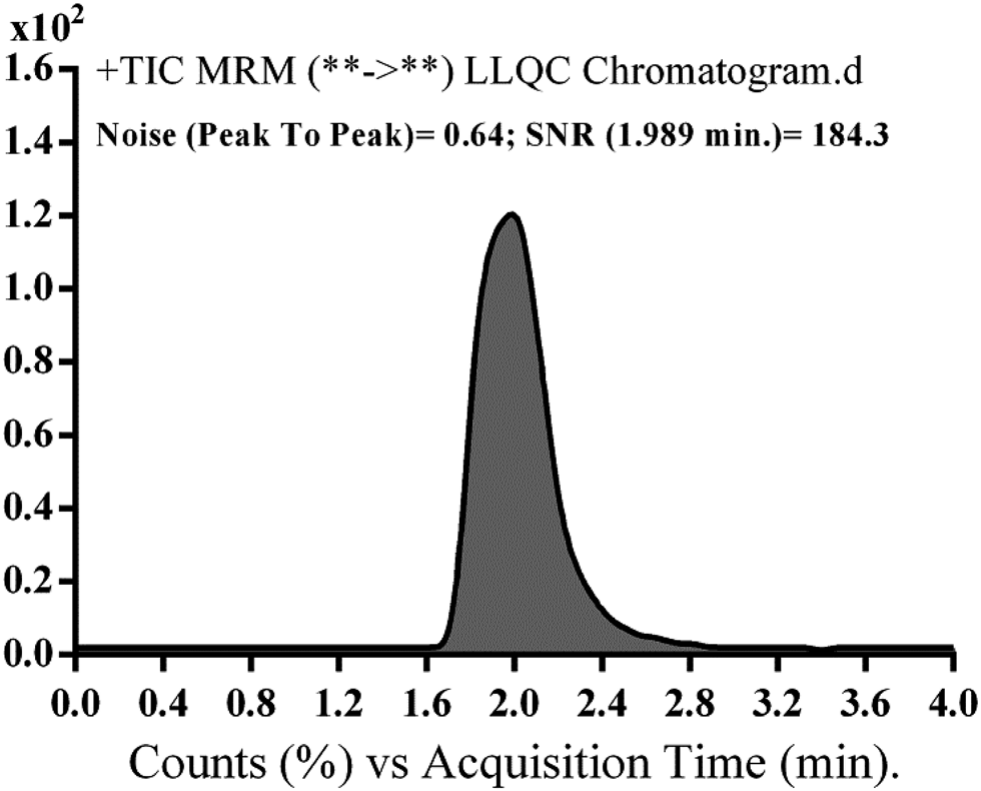
Naquotinib lower limit quality control (LLQC) multiple reaction monitoring (MRM) chromatogram revealed high signal to noise (S/N) ratio.

The range of linearity and *r*^2^ for the supposed method were 5–500 ng mL^−1^ and ≥0.9999, respectively, in the HLM matrix. The regression equation of the NQT calibration curve was *y* = 1.5581*x* − 3.395. The LOD and LOQ were 0.78 and 2.36 ng mL^−1^, respectively, while the LLQC peak showed a high signal to noise (S/N) ratio and a good peak shape, suggesting the sensitivity of the developed LC-MS/MS method (Fig. 5).

**Fig. 6:**
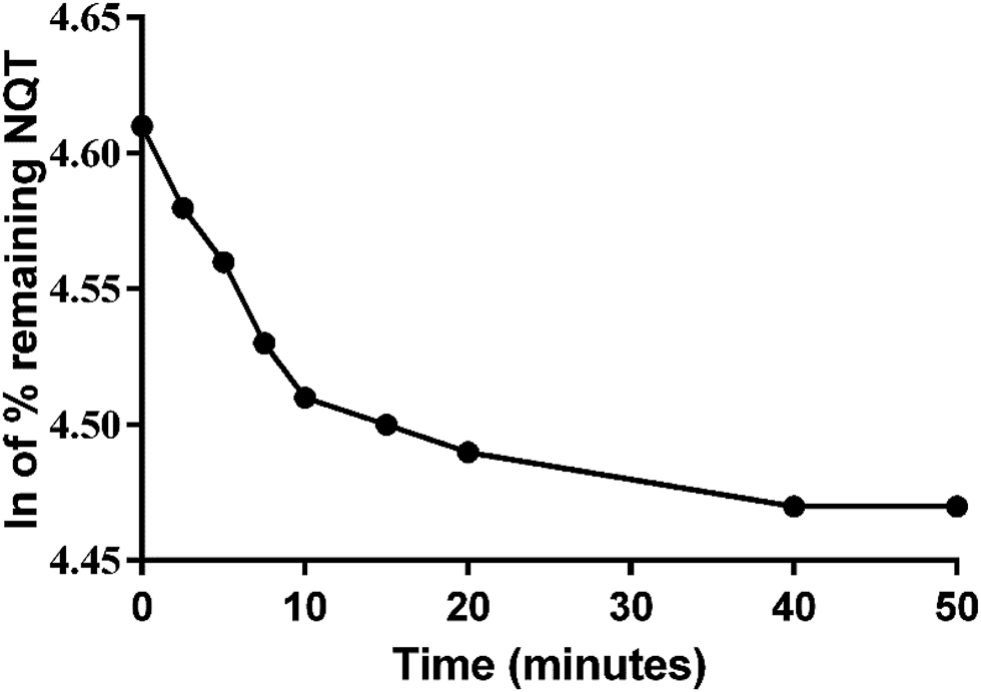
Metabolic stability curve of naquotinib (NQT) in human liver microsomes (HLMs).

The RSD values of the six repetitions of each calibration level were <1.96% in the HLM matrix (Table 2). Back calculations for the 12 NQT standards in the HLM matrix (calibration and QC standards) confirmed the performance of the established method. The intra-day and inter-day precisions and accuracies were 0.99 to 2.58% and −6.36 to 1.88, respectively (Table 3). The average NQT recoveries were 98.61 ± 2.42% in the HLM matrix (Fig. 6).

**Table 2:** Naquotinib (NQT) back-calculated calibration standard concentrations from human liver microsome (HLM) matrix

NQT Nominal concentrations (ng mL^−1^)	Mean^a^	SD	RSD (%)	Accuracy (%)
5 (LLQC)	5.28	0.08	1.52	−5.65
10	10.29	0.21	2.07	−2.95
15 (LQC)	14.85	0.23	1.52	1.00
30	28.66	0.60	2.10	4.46
50	50.18	0.76	1.51	−0.36
80	78.07	2.09	2.68	2.42
100	100.94	1.75	1.74	−0.94
150 (MQC)	148.18	2.49	1.68	1.21
200	201.63	2.28	1.13	−0.82
300	294.62	2.20	0.75	1.79
400 (HQC)	397.32	2.76	0.70	0.67
500	496.02	5.79	1.17	0.80

a Mean of six replicates; RSD, relative standard deviation.

**Table 3:** Intra-day and inter-day (accuracy and precision) of established method^a^

HLM matrix	LLQC (5 ng mL^−1^)		LQC (15 ng mL^−1^)		MQC (150 ng mL^−1^)		HQC (400 ng mL^−1^)	
	Intra-day assay^b^	Inter-day assay^c^	Intra-day assay	Inter-day assay	Intra-day assay	Inter-day assay	Intra-day assay	Inter-day assay
Mean	5.28	5.32	14.85	14.75	148.18	147.18	397.32	393.32
SD	0.08	0.05	0.23	0.38	2.49	2.71	2.76	6.70
Precision (%RSD)	1.52	0.99	1.52	2.58	1.68	1.84	0.70	1.70
% accuracy	−5.65	−6.36	1.00	1.69	1.21	1.88	0.67	1.67

a HLM, human liver microsome; LLQC, lower limit quality control; LQC, low quality control; MQC, medium quality control; HQC, high quality control.

b Mean of twelve replicates on the same day.

c Mean of six replicates for three days.

#### 3.2.3. Precision and accuracy

As can be seen in Table 3, the values of the intra- and inter-day accuracy and precision are acceptable according to FDA guidelines.^24^

#### 3.2.4. Matrix effects and extraction recovery

Table 4 shows the percentage recovery of the QC samples for computing the NQT level in the HLM matrix. The NQT and FTB recoveries were 98.61 ± 2.42 and 98.7 ± 0.7% in the HLM matrix, respectively. The absence of a matrix effect on NQT or FTB was proven by analyzing two batches of HLMs, which were spiked with NQT LQC and FTB (15 and 50 ng mL^−1^, respectively). These batches were marked as set 1. Set 2 was prepared using the mobile phase in place of the HLM matrix and, thus, the matrix effect was calculated using the following equation:
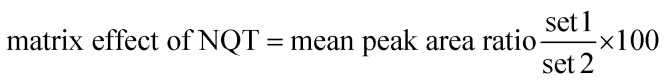

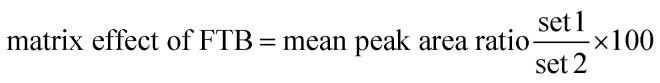


**Table 4:** Recovery of naquotinib (NQT) samples in human liver microsome (HLM) matrix

Conc. (ng mL^−1^)	HLM matrix			
	5 ng mL^−1^	15 ng mL^−1^	150 ng mL^−1^	400 ng mL^−1^
Mean^a^	5.09	14.85	143.89	391.34
SD	0.05	0.38	2.56	5.40
Precision (RSD%^b^)	0.99	2.58	1.78	1.38
Recovery (%)	101.71	98.98	95.93	97.83
FTB recovery	98.7 ± 0.7%			

a Mean of six replicates.

b RSD, relative standard deviation.

The studied HLMs containing NQT and FTB showed matrix effects of 98.61 ± 2.42 and 98.7 ± 0.7%, respectively. The internal standard normalized matrix effect (IS normalized MF) was computed using the following equation:
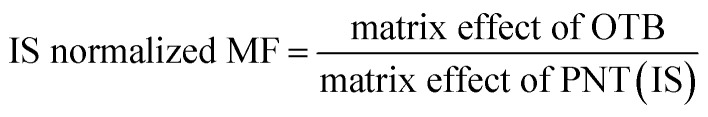


The IS normalized MF was 1.01 and it was within the range of adequacy.^25^ Accordingly, these results showed that the HLM matrix had no obvious influence on the ionization of NQT or FTB.

### 3.3. Metabolic stability

The NQT concentration in the HLM matrix was calculated by displacing the peak area ratios in the calibration curve regression equation. The metabolic stability curve was constructed by plotting the ln NQT percentage remaining on the *y*-axis against the time of incubation on the *x*-axis (Fig. 5). The linear part of the regression equation was used for computing *in vitro t*_1/2_.^26^ The regression equation was *y* = −0.0102*x* + 4.6069 with *r*^2^ = 0.9981 (Table 5).

**Table 5:** Metabolic stability parameters of naquotinib (NQT) incubated with human liver microsomes (HLMs) for specific time intervals

NQT metabolic stability parameters				
Time (min)	Conc. (ng mL^−1^)	*X* a	Parameter	Value
0	446.22	4.61	Regression equation^b^	*y* = −0.0102*x* + 4.6069
2.50	436.70	4.58		
5.00	424.60	4.56	*r* ^2^ c	0.9981
7.50	413.26	4.53		
10.00	403.56	4.51	Slope	0.0102
15.00	399.70	4.50		
20.00	396.31	4.49	*t*_1/2_ d	67.96 min
40.00	391.25	4.47		
50.00	390.14	4.47	Cl_int_^e^	2.12 mL min^−1^ kg^−1^
90.00	387.01	4.46		

a *X*: ln of percentage of NQT remaining.

b Linear part regression equation.

c Correlation coefficient.

d Half-life.

e Intrinsic clearance.

Using the following equations:
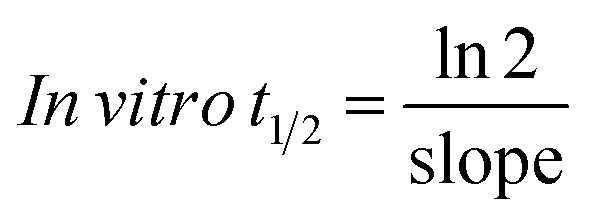
the slope was calculated to be 0.0044.
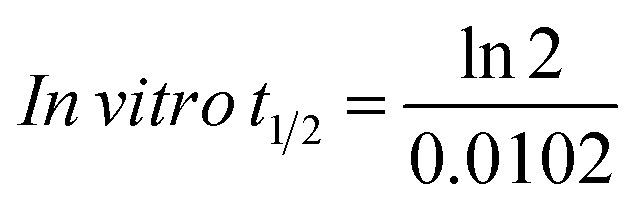
*In vitro t*_1/2_ = 67.96 min

The intrinsic clearance of NQT was computed using the *in vitro t*_1/2_ method^17^ using the following equation:
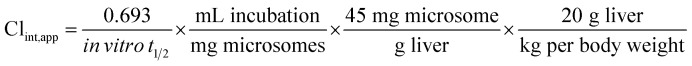

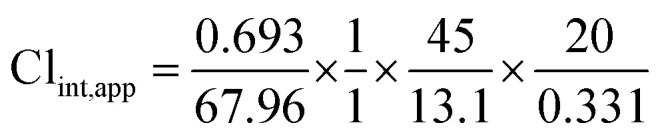
Cl_int, app_ = 2.12 mL min^−1^ kg^−1^

These results revealed that the metabolic stability of NQT was characterized by a very low Cl_int_ (2.12 mL min^−1^ kg^−1^) and very long *in vitro t*_1/2_ value (67.96 min) that revealed the moderate clearance of NQT from the blood by the liver and possible well-controlled *in vivo* bioavailability. This drug could be utilized without accumulation inside the body or fast elimination from the blood.^27^

## 4. Conclusions

An LC-MS/MS method was established and validated for quantifying NQT. The established method exhibited good sensitivity, was eco-friendly with the small volume of ACN used, fast, accurate, and showed high recovery. The LC-MS/MS method was utilized for NQT metabolic stability assessment in an HLM matrix using two parameters: *in vitro t*_1/2_ and Cl_int_. These two outcomes indicate that NQT showed moderate clearance from the blood by the liver and possible well-controlled *in vivo* bioavailability.^28–30^ Therefore, this drug could be utilized without concerns about accumulation inside the body or fast elimination from the blood.

## Conflicts of interest

The authors declare no conflict of interest.

## Abbreviations

CE: Collision energy
EGFR: Epidermal growth factor receptor
FV: Fragmentor voltage
HLMs: Human liver microsomes
HQC: High quality control
IS: Internal standard
IS normalized MF: Internal standard normalized matrix effect
LC-MS/MS: Liquid chromatography tandem mass spectrometry
LOD: Limit of detection
LQC: Lower quality control
LOQ: Limit of quantification
MQC: Medium quality control
NSCLC: Non-small cell lung cancer
NQT: Naquotinib
FTB: Foretinib
QC: Quality control
TKI: Tyrosine kinase inhibitor
RRLC: Rapid resolution liquid chromatography

## Supplementary Material
